# Diuretics Prime Plant Immunity in *Arabidopsis thaliana*

**DOI:** 10.1371/journal.pone.0048443

**Published:** 2012-10-29

**Authors:** Yoshiteru Noutoshi, Mika Ikeda, Ken Shirasu

**Affiliations:** 1 Research Core for Interdisciplinary Sciences (RCIS), Okayama University, Kita-ku, Okayama, Japan; 2 RIKEN Plant Science Center, Tsurumi, Yokohama, Japan; University of Wisconsin-Milwaukee, United States of America

## Abstract

Plant activators are agrochemicals that activate the plant immune system, thereby enhancing disease resistance. Due to their prophylactic and durable effects on a wide spectrum of diseases, plant activators can provide synergistic crop protection when used in combination with traditional pest controls. Although plant activators have achieved great success in wet-rice farming practices in Asia, their use is still limited. To isolate novel plant activators applicable to other crops, we screened a chemical library using a method that can selectively identify immune-priming compounds. Here, we report the isolation and characterization of three diuretics, bumetanide, bendroflumethiazide and clopamide, as immune-priming compounds. These drugs upregulate the immunity-related cell death of *Arabidopsis* suspension-cultured cells induced with an avirulent strain of *Pseudomonas syringae* pv. *tomato* in a concentration-dependent manner. The application of these compounds to *Arabidopsis* plants confers disease resistance to not only the avirulent but also a virulent strain of the pathogen. Unlike salicylic acid, an endogenous phytohormone that governs disease resistance in response to biotrophic pathogens, the three diuretic compounds analyzed here do not induce *PR1* or inhibit plant growth, showing potential as lead compounds in a practical application.

## Introduction

Pesticides such as microbicides, fungicides and insecticides are widely used to cover the demand for the expanding world population and biomass [1]. Even with pesticides, the total crop loss from disease is estimated to be approximately 14% of the total potential production ability of the planet. Importantly, the percentage of crop loss due to disease and insects has not changed over the last 30 years [2]. In addition, the effects of pesticides are often overcome by spontaneous mutations in the pathogenic organisms. Many pesticides are associated with risks to both the environment and food production. Furthermore, pesticides often affect not only the targeted organisms but also useful predators or antagonistic microorganisms.

In contrast to the agrochemicals that target pests, plant activators work in plants and increase their ability to resist pests [3]. For example, the most successful plant activator, probenazole, was originally developed 35 years ago [4]. Probenazole has been widely used in the cultivation of irrigated rice in East Asia to protect against blast fungus and bacterial leaf blight. The remarkable advantage of probenazole is its durability, and indeed, no drug-resistant pathogens have emerged, most likely because it acts on host crops but not pathogens. Interestingly, probenazole potentiates but does not directly induce the defense responses in plants. Probenazole stimulates the accumulation of salicylic acid (SA), a defense hormone in plants, but its target and its precise mode of action remain unclear [5]. In contrast, benzothiadiazole (BTH), another practical plant activator, functions as a synthetic chemical analog of SA [6], [7]. BTH and SA effectively mount a disease resistance response in plants. However, the application of BTH has been confined to specific crops, mainly due to accompanying drug injuries at high dosages. These injuries are most likely due to the strong induction of the defense response, which is often associated with severe growth suppression [8], [9], [10], [11].

To obtain novel lead compounds, we established a method for selectively identifying agents that enhance but do not induce the disease resistance response in plants [12]. The method was miniaturized for high-throughput screening to explore chemical libraries that contain a large number of compounds. From a comprehensive survey of a commercially available library of diversity-oriented small molecules, we have successfully identified several compounds that prime the immune response in *Arabidopsis* plants and have investigated five of these compounds, which was designated as imprimatinA1, A2, A3, B1 and B2 [12]. In this study, we screened an additional chemical library containing pharmaceutical drugs and natural products, and identified three diuretics as plant immune system-priming compounds. These compounds potentiate but do not directly induce the defense responses and show potential as lead structures for novel plant activators.

## Results

### Isolation of three diuretics as plant immune-priming compounds

**Figure 1:**
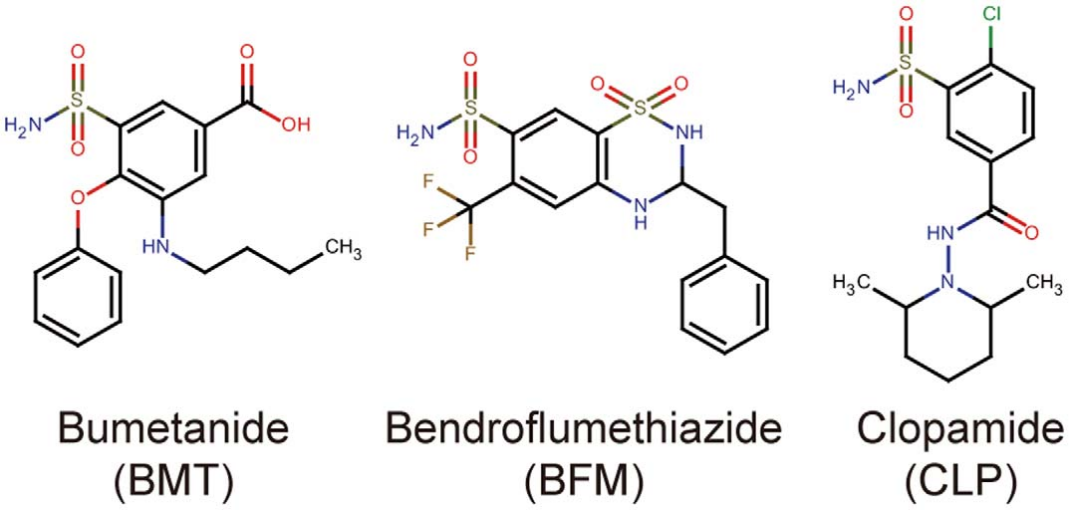
Molecular structures of the isolated diuretics as plant immune-priming compounds.

Using a previously established high-throughput chemical screening method for plant immune-priming agents [12], we searched for chemical compounds that enhance cell death in cultured *Arabidopsis* cells after infection with an avirulent pathogenic bacteria, *Pseudomonas syringae* pv. *tomato* DC3000 (*Pst*) *avrRpm1*. We used a commercially available chemical library comprised of 2,000 small organic molecules, including known pharmaceutical drugs, experimental bioactive compounds and natural products (MicroSource Discovery Systems Inc.). After performing an initial screen made up of three replicates, the concentration dependency of the candidate chemicals was analyzed. The candidate compounds that reproducibly enhanced cell death were classified into different groups based on both their pharmaceutical properties and their molecular structures. For this study, we focused on a group containing three different diuretics, bumetanide, bendroflumethiazide and clopamide (Fig. 1).

**Figure 2:**
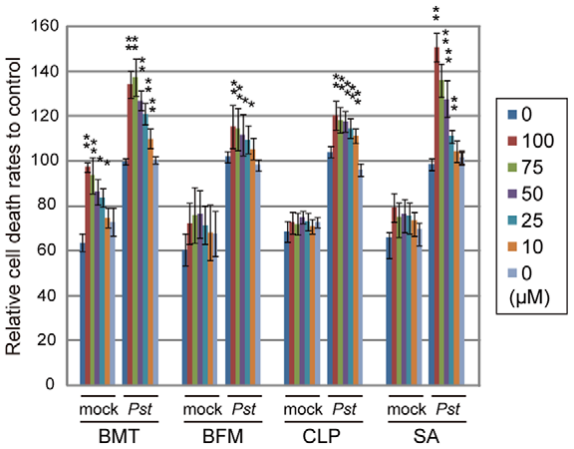
The effect of the diuretics on the pathogen-induced cell death of *Arabidopsis* suspension cells. *Arabidopsis* suspension cultured cells were incubated with the compounds with or without the avirulent bacterial pathogen *Pseudomonas syringae* pv. *tomato* DC3000 (*Pst*) *avrRpm1*, and the extent of cell death was quantitatively measured by the concentration of Evans blue dye. Each cell death rate is shown as a value relative to the mean of a mock treatment with the pathogen used for each experimental group. Sodium salicylate (SA) was used as a positive control. Error bars represent ±SE of four independent replicates. ** : *P*<0.01, * : *P*<0.05; Student's *t*-test with post-hoc Bonferroni's correction.

*Arabidopsis* suspension cells were treated with these diuretics at different concentrations, and the rates of cell death were quantitatively measured after inoculation with *Pst-avrRpm1*. These compounds upregulated the cell death induced by the pathogen in a concentration-dependent manner (Fig. 2). Their effects on cell death ranged from half to one-third of SA, which can function as an endogenous potentiator for pathogen-triggered cell death [13]. Bumetanide appeared to have a stronger effect than the other diuretics, however, it would be due to its concentration-dependent toxic effects on the cells in the absence of the pathogens (Fig. 2). This chemical activity is similar to those of ImprimatinA1, A3, B1 and B2 which were the plant activators isolated from our previous screening and the commercial plant activator tiadinil [12]. Therefore, we further analyzed these compounds including BMT.

### The diuretics increased disease resistance in Arabidopsis plants

**Figure 3:**
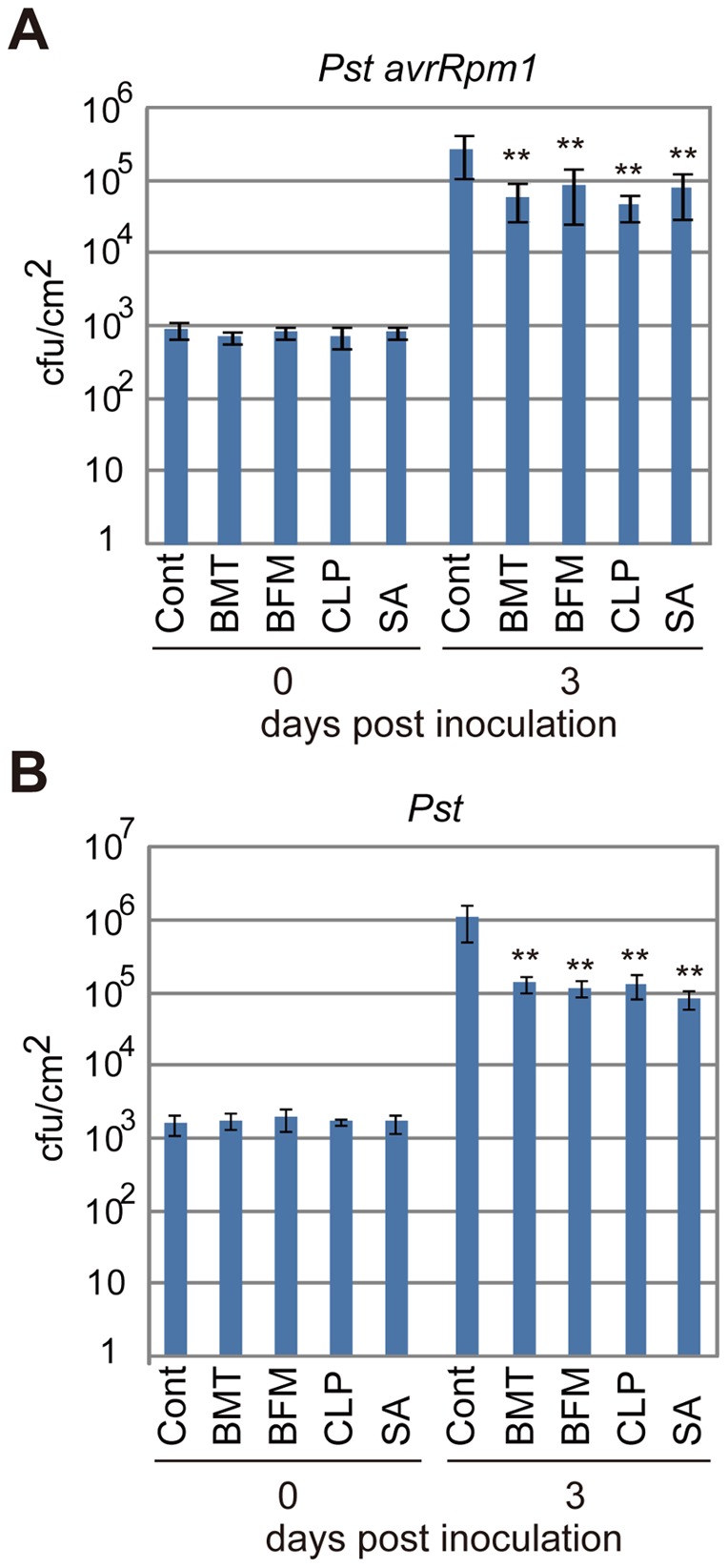
The increased disease resistance induced by the diuretics in *Arabidopsis* plants. *Arabidopsis* seedlings were grown on rockwool for three weeks under short-day conditions, and their roots were drenched with water supplemented with 100 µM of the compounds for three days. As a positive control, 50 µM of sodium salicylate (SA) was used. Then, the avirulent *Pst-avrRpm1* (A) and the virulent *Pst* (B) were inoculated into the leaves by syringe infiltration, and the bacterial numbers were counted at the indicated days (n = 4). **P<0.01; Student's *t*-test.

**Figure 4:**
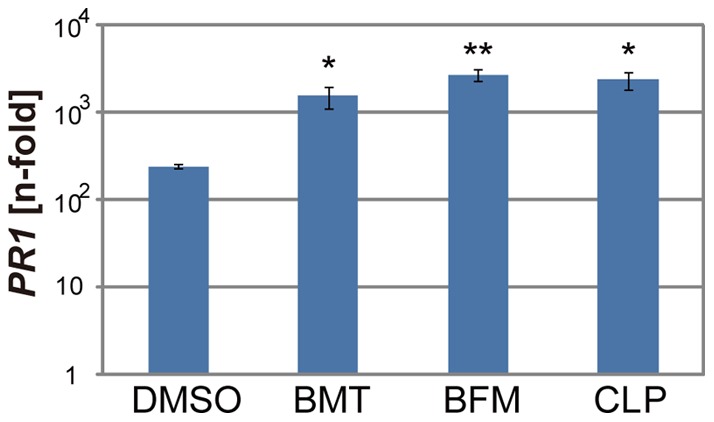
The expression of *PR1* gene 24 hours after pathogen inoculation with or without the application of the diuretics. The *PR1* transcript level was determined by qRT-PCR. cDNA were prepared from seedlings 24 hours after inoculation of *Pst-avrRpm1* with or without 100 µM of the diuretics. DMSO was used as mock treatment. The expression values were normalized to *Actin2* as an internal standard. The bar represents the SE of three independent replicates. **P<0.01, *P<0.05; Student's *t*-test.

**Figure 5:**
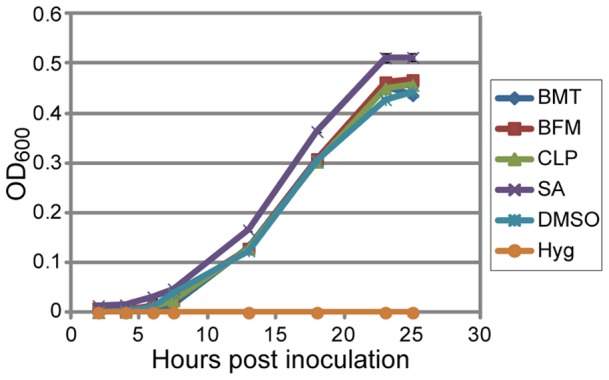
Effect of the diuretics on the growth of pathogenic bacteria. *Pst* was cultured in liquid minimal medium supplemented with 200 µM of the indicated chemicals or 100 µg/mL hygromycin, and bacterial growth was monitored as the optical density of the bacteria at 600 nm at the indicated times after inoculation. The bar represents the SE of three independent replicates.

Next, we examined if these diuretics functioned in plants. *Arabidopsis* seedlings were grown hydroponically on rockwool, and their roots were immersed in water solutions containing each diuretic at 100 µM. SA and DMSO were applied at 50 µM as a positive and negative control, respectively. Three days after incubation, a virulent and an avirulent *Pst* strains were inoculated into the leaves, and the numbers of bacteria inside the leaves were counted at the indicated days post inoculation (DPI). All chemicals significantly decreased the growth of avirulent bacteria compared with the mock control experiment (Fig. 3a). Interestingly, the diuretic treatments also suppressed the growth of the virulent *Pst* (Fig. 3b). The levels of enhanced resistance to both the avirulent and virulent *Pst* were almost the same as those of 50 µM SA (Figs. 3a and 3b). The effects of these diuretics on disease resistance *in planta* were consistent with those on the pathogen-induced cell death of the suspension cells (Fig. 2). According to the upregulation of the disease resistance in the diuretics-treated *Arabidopsis* plants, the greater levels of *PR1* gene expression were detected at 24 hours after infection of *Pst* (Fig. 4). We also tested if the diuretics had inhibitory effects on bacterial growth and found that these compounds did not prevent bacterial growth at a concentration of 200 µM (Fig. 5).

### The diuretics did not directly induce PR1 unlike SA

**Figure 6:**
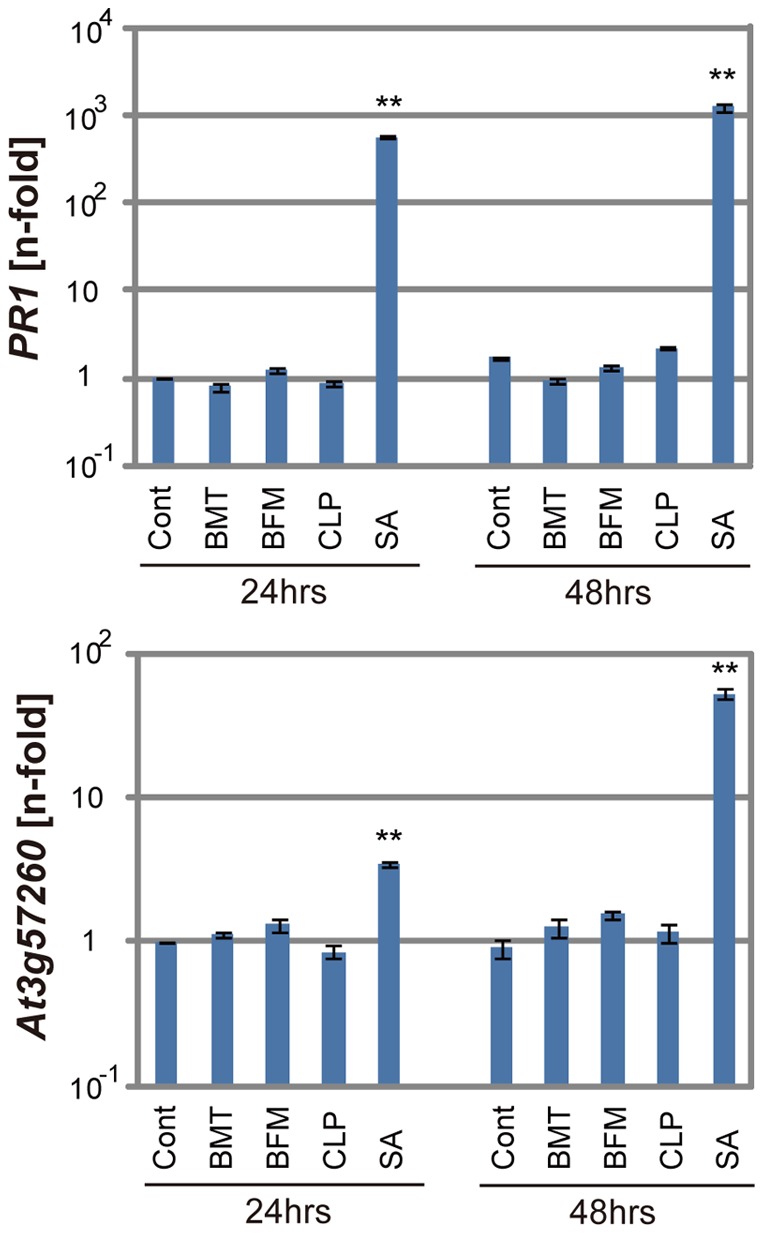
The expression of defense genes after the application of the compounds. The mRNA transcript levels of *PR1* and *At3g57260* were determined by qRT-PCR using cDNA prepared from 10-day-old seedlings soaked in liquid media containing 100 µM of the chemicals for 24 or 48 hours. The expression values of the individual genes were normalized to *Actin2* as an internal standard. These results are representative of three independent replicates. **P<0.01; Student's *t*-test.

Next, we examined if these diuretics possess activity analogous to that of SA. Young *Arabidopsis* seedlings were treated with the diuretic compounds for 24 or 48 hours at 100 µM, and the expression of defense genes was analyzed. The transcription levels of the *pathogenesis-related 1* (*PR1*) gene, a marker of SA-dependent defense responses, and *A3g57260*, an SA-inducible gene [14], were examined by real-time quantitative reverse transcriptase polymerase chain reaction (qRT-PCR). Although SA effectively induced the expression of these genes at both time points, none of the examined diuretics induced these genes (Fig. 6). These results suggest that the tested diuretics and SA function differently.

### The diuretics did not inhibit SA glucosyltransferase

**Figure 7:**
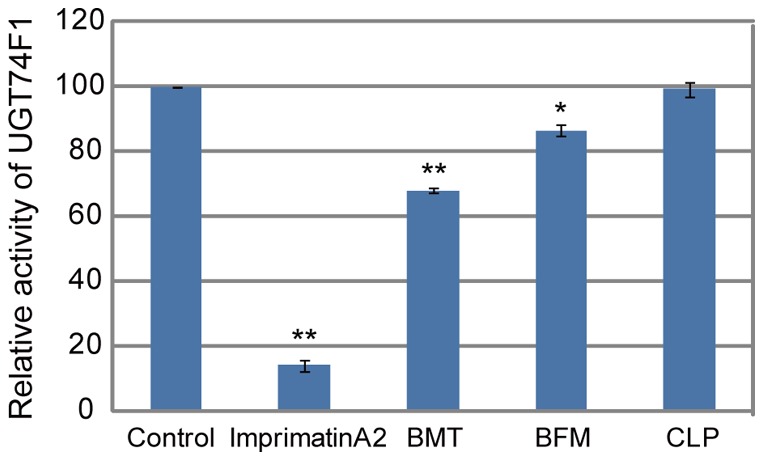
Effect of the diuretics on the salicylic acid glucosyltransferase activity of UGT74F1. The production levels of SA-β-D-glucoside (SAG) were measured by HPLC in the *in vitro* enzymatic reaction supplemented with 100 µM of the compounds using affinity-purified histidine-tagged recombinant UGT74F1 protein expressed in *E. coli* [12]. Imprimatin A2 was used as a positive control. The data shown are relative to the DMSO control. The error bars represent the SE of independent triplicates. **P<0.01, *P<0.05; Student's *t*-test.

In the previous study, we revealed that the perturbation of SA glucosylation, a major metabolic pathway used during defense responses, is one of the modes of action for plant immune-priming agents [12]. Therefore, we examined whether the isolated diuretics inhibit the enzymatic activity of SA glucosyltransferase (SAGT). Each diuretic was added to an *in vitro* reaction mixture containing UGT74F1, one of the *Arabidopsis* SAGTs, and their effects were evaluated. ImprimatinA2, a positive control, clearly inhibited the SAGT activity, whereas clopamide had no inhibitory effects on the SAGT enzymatic activity at 100 µM (Fig. 7). Considering that Imprimatins had more than 85% inhibitory activities of the *Arabidopsis* SAGTs at 100 µM [12], 10–30% inhibitions of the SAGT activity of BMT and BFM were likely to be insufficient to account for their priming effects on plant immunity.

### The diuretics did not inhibit plant growth

**Figure 8:**
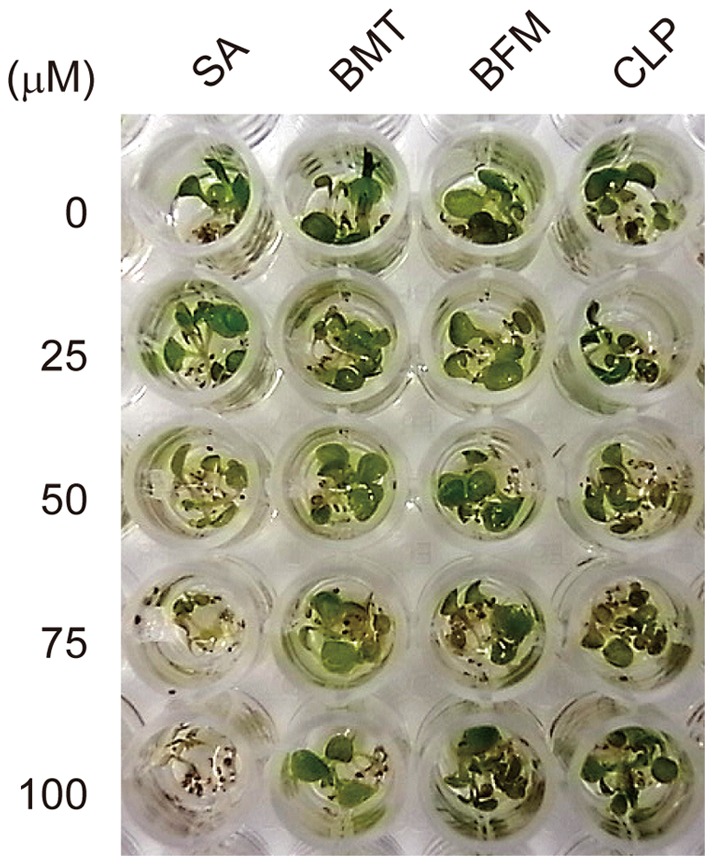
The effect of the diuretics on the germination and growth of *Arabidopsis thaliana*. *Arabidopsis* seeds were dispensed into each well of a 96-well plate, and liquid media containing each chemical at the indicated concentrations was applied. Then, the plate was placed under long-day conditions at 22°C, and a photograph was taken after two weeks.

To evaluate the potential of these diuretics for practical applications as plant activators, the effects of the compounds on plant growth were examined. After sterilization and stratification, *Arabidopsis* seeds were incubated with liquid media containing each of the diuretics at varied concentrations. In contrast to SA, which inhibited the growth of *Arabidopsis* seedlings in a concentration-dependent manner, the tested compounds did not severely prevent germination or growth at an effective concentration range for the enhancement of cell death and disease resistance (Fig. 8). Consistent with the toxicity effect of BMT on the suspension cells (Fig. 2), it seems to have some negative effect on germination/growth of *Arabidopsis* seedlings (Fig. 8).

## Discussion

In this study, a high-throughput chemical screening method was used to isolate three diuretics, bumetanide, bendroflumethiazide and clopamide, that act as plant immune-priming compounds. These compounds enhanced the pathogen-induced cell death of suspension-cultured *Arabidopsis* cells in a concentration-dependent manner (Fig. 2). The application of the diuretics to *Arabidopsis* seedlings enhanced disease resistance (Fig. 3), likely due to the immune-priming effects of the compounds, which is supported by the absence of any toxic effects on pathogenic bacteria (Fig. 5). Thus, the screen using the suspension-cultured cells is suitable for identifying lead compounds for priming plant immunity.

Consistent with our screening strategy, the isolated compounds did not induce the two tested defense genes in the absence of pathogens (Fig. 6). Likewise, these compounds did not prevent germination and plant growth during the early seedling stage (Fig. 8). Compared with other screening methods, which use activation potency of defense gene expression as an indicator, our screening method can provide unique lead compounds with less phytotoxic effects and is thus suitable for practical use.

In addition to being lead compounds for crop protectants, the diuretics can also be useful probes for understanding plant immunity at the molecular level. We demonstrated that these diuretics do not function as analogs of SA, unlike BTH and INA (Fig. 6). Furthermore, they did not inhibit the SA glucosylation enzyme that was previously identified as a target of other immune-priming compounds [12] (Fig. 7). These results imply that these diuretics potentiate plant immunity by an unknown mechanism.

Diuretics are pharmaceutical drugs used in clinical practice to treat hypertension or edema [15]. Diuretics target the sodium-chloride symporters, a type of electroneutral cation-coupled chloride cotransporter known as SLC12 (solute carrier family 12), which are broadly conserved in the animal kingdom [16]. Diuretics act on the SLC12A family members, which are sodium (Na^+^)-coupled chloride (Cl^−^) cotransporters located along the renal tubule of the kidney nephron. By binding to the Cl^−^ binding site of the SLC12A proteins and competing with Cl^−^ [16], diuretics inhibit Na^+^ and Cl^−^ reabsorption from the tubular fluid into the cells. This interferes with urine concentration and dilution [15]. In the *Arabidopsis* genome, only a single protein, AtCCC1/HAP5 (At1g30450), has a high similarity to animal cation-Cl^−^ cotransporters (CCC) such as SLC12A [17]. AtCCC1 was shown to have Cl^−^ transport activity that depended on both Na^+^ and K^+^ using a cRNA expression system with *Xenopus laevis* oocytes. Interestingly, the Cl^−^ uptake activity of AtCCC1 was also demonstrated to be reduced in the presence of 100 µM bumetanide *in vitro* [17]. Thus, the inhibition of AtCCC1 might be a potential target of the isolated diuretics for cell death and disease resistance in *Arabidopsis*. However, the target of each of the diuretics is different in animals, although their inhibitory mechanism is shared. For instance, bumetanide, a loop diuretic, targets the Na^+^-K^+^-2Cl^−^ cotransporters SLC12A1 and SLC12A2 [15], [16], whereas bendroflumethiazide, a thiazide diuretic, and clopamide, a thiazide-like diuretic, act on the Na^+^-Cl^−^ cotransporter SLC12A3 [15], [16]. In our experiments, the activities of these diuretics are nearly the same in priming plant immunity (Figs. 2 and 3). This situation suggests the possibility that these diuretics might act on other proteins to potentiate plant immunity.

Some diuretics are known to have the potential to cause a hypersensitive allergic reaction when used as medication, and this effect is thought to be derived from the sulfonamide groups of the diuretics [18]. Accordingly, the isolated diuretics in this study have molecular structures containing the sulfonamide functional group (Fig. 1). Plants do not have allergic reaction, however, the immune-priming effect of the diuretics might be dependent on this sulfonamide moiety with unknown mechanism. The treatment of *Arabidopsis* seedlings with the sulfonamide compounds, sulfamethoxazole, sulfadiazine and sulfapyridine, decreased the growth of pathogenic bacteria inside of the plants [19]. Sulfamethoxazole had the strongest effect among the tested sulfonamides, and it can confer disease resistance in soil-grown *Arabidopsis* seedlings at 100 µM. The effective concentration range of these sulfonamides is very similar to those of the diuretics. The chemical library used in this study contains ethacrynic acid, spironolactone and triamterene, which are diuretics without a sulfonamide group. None of these non-sulfonamide diuretic compounds were identified as positives in our screen, although ethacrynic acid targets Na^+^-K^+^-2Cl^−^ cotransporters and is a loop diuretic [20]. Further analysis of the mode of action of the diuretics or sulfonamides will reveal new insights into plant immunity and provide a novel tool for conferring disease tolerance in plants.

## Materials and Methods

### Chemicals

A commercial chemical library of the Spectrum Collection containing 1,000 medical drugs, 500 natural products with unknown biological properties and 420 non-drug bioactive compounds was purchased (The Spectrum Collection, 10 mM in DMSO, MicroSource Discovery System Inc., Gaylordsville, CT, USA). The prohibited imports in the library were excluded. Compounds were further obtained from the chemical companies indicated below. Bumetanide, bendroflumethiazide and sodium salicylate were purchased from Sigma-Aldrich (St Louis, MO, USA). Clopamide was purchased from BIOMOL International (Plymouth meeting, PA, USA).

### Plant materials and growth conditions

*Arabidopsis* suspension-cultured cells were grown in liquid media containing MS with 3% sucrose supplemented with 0.5 mg/L MES (pH 5.7), 0.5 mg/L naphthaleneacetic acid and 0.05 mg/L 6-benzylaminopurine under long-day conditions (16-h light/8-h dark cycles) [21], [22]. For the gene expression analysis, *Arabidopsis thaliana* ecotype Columbia was grown on half-MS agar media (1% sucrose) at 22°C under long-day conditions. To assay for disease resistance, the plants were grown in soil at 22°C under short-day conditions (8-h light/16-h dark cycles). For the growth assay, *Arabidopsis* seeds were sterilized and stored at 4°C for 4 days to break dormancy. Then, the seeds were dispensed into 96-well plates, and half-MS liquid media supplemented with 1% sucrose containing each chemical was applied at the indicated concentrations. The plates were then incubated at 22°C under long-day conditions.

### Quantitative measurement assay for the chemical's effect on cell death

The method was performed essentially as described previously [12]. *Arabidopsis* suspension cells were dispensed into each well of 96-well plates, and the chemicals were added at the indicated concentrations. After a one hour incubation with occasional tapping, *Pseudomonas syringae* pv. *tomato* DC3000 (*Pst*) *avrRpm1* (final concentration: OD = 0.2 in MS medium) and MgCl_2_ as mock were applied to each duplicated well, respectively. After co-cultivation on a shaker for 21 hours under long-day conditions at 22°C, the cells were stained with 1% Evans blue dye. Then, the cells were washed with 1 mL of water four times, and the incorporated dye was eluted with 400 µL of elution solution (50% methanol, 1% SDS). The absorbance at 595 nm was measured with a microplate reader and was taken as the degree of cell death. The cell death rates were calculated as the value relative to the mock control at 100%. The data are represented as the mean ± SD (n = 5).

### Chemical treatment of plants and RNA experiments

*Arabidopsis* seedlings grown on agar media for two weeks were soaked in liquid half-MS media (1% sucrose) supplemented with 100 µM of the chemicals. The plants were incubated for 24 or 48 hours at 22°C. The seedlings were collected in a 2-mL tube and frozen in liquid nitrogen. Then, the samples were crushed with four zirconia balls (*ø* 2 mm) using the Shake Master Neo (BMS, Tokyo, Japan). Total RNA was extracted with the PureLink Micro-to-Midi Total RNA Purification System with on-column DNase treatment (Invitrogen, Carlsbad, CA, USA). The RNA concentration and purity were validated by a spectrometer (BioPhotometer plus; Eppendorf, Germany). cDNA was synthesized from each sample with the PrimeScript RT reagent kit with gDNA Eraser (Perfect Real Time) (Takara, Shiga, Japan). The qRT-PCRs were performed using the KAPA SYBR Fast qPCR Kit (KAPA BIOSYSTEMS, Woburn, MA, USA) with a LightCycler® 480 real-time PCR instrument (Roche Diagnostics, Basel, Switzerland). The quantitation of the target transcript was conducted using the LightCycler 480 internal software “Absolute Quantification 2^nd^ Derivative Max” and normalized to *Actin2*. The primers used in this study were 5′-CCGCTCTTTCTTTCCAAGC-3′ and 5′-CCGGTACCATTGTCACACAC-3′ for *Actin2*, 5′-TGATCCTCGTGGGAATTATGT-3′ and 5′-TGCATGATCACATCATTACTTCAT-3′ for *PR1* and 5′- CTTAGCCTCACCACCAATGTTG-3′ and 5′- TCCCGTAGCATACTCCGATTTG-3′ for *At3g57260*.

### Assay for disease resistance

The inoculation and assessment of bacterial growth in the plants was performed essentially as previously described [23]. *Arabidopsis* seedlings grown on MS agar plates for one week under short-day conditions were transferred onto rockwool and hydroponically cultivated at 22°C for 3 weeks. Then, the plants grown on rockwool were transferred to a small pot, and water supplemented with 100 µM of the chemicals was supplied. The plants were then grown for 3 days prior to the inoculation with the pathogenic bacteria. As a positive control, 50 µM of SA was used. Freshly grown bacteria of both the *Pst* and *Pst avrRpm1* strains resuspended in 10 mM MgCl_2_ to an OD_600_ of 0.002 were inoculated into leaves with a needleless syringe. After 3 days, leaf disks were punched out with a sterile cork-borer and placed in a 2-mL tube. Then, the disks were crushed with zirconia balls in 500 µL of 10 mM MgCl_2_. A serial dilution of each sample was spread onto agar media containing kanamycin and rifampicin, and the number of bacteria inside the leaves was calculated.
